# Research on the Strengthening Advantages on Using Cellulose Nanofibers as Polyvinyl Alcohol Reinforcement

**DOI:** 10.3390/polym12040974

**Published:** 2020-04-22

**Authors:** Quim Tarrés, Helena Oliver-Ortega, Manel Alcalà, F. Xavier Espinach, Pere Mutjé, Marc Delgado-Aguilar

**Affiliations:** 1 LEPAMAP Research Group, Department of Chemical and Agricultural Engineering and Agrifood Technology, University of Girona, Maria Aurèlia Capmany, 61-17003 Girona, Spain; joaquimagusti.tarres@udg.edu (Q.T.); helena.oliver@udg.edu (H.O.-O.); pere.mutje@udg.edu (P.M.); 2 PRODIS Research Group, Department of Organization, Business Management and Product Design, University of Girona, Maria Aurèlia Capmany, 61-17003 Girona, Spain; manuel.alcala@udg.edu (M.A.); francisco.espinach@udg.edu (F.X.E.)

**Keywords:** cellulose nanofibers, polyvinyl alcohol, nanocomposites, nanocellulose, natural fiber reinforced composites

## Abstract

The present work aims to combine the unique properties of cellulose nanofibers (CNF) with polyvinyl alcohol (PVA) to obtain high-performance nanocomposites. CNF were obtained by means of TEMPO-mediated ((2,2,6,6-Tetramethylpiperidin-1-yl)oxyl) oxidation, incorporated into the PVA matrix by means of compounding in a single-screw co-rotating internal mixer and then processed by means of injection molding. It was found that CNF were able to improve the tensile strength of PVA in 85% when 4.50 wt % of CNF were added. In addition, the incorporation of a 2.25 wt % of CNF enhanced the tensile strength to the same level that when 40 wt % of microsized fibers (stone groundwood pulp, SGW) were incorporated, which indicated that CNF possessed significantly higher intrinsic mechanical properties than microsized fibers. SGW was selected as reference for microsized fibers due to their extended use in wood plastic composites. Finally, a micromechanical analysis was performed, obtaining coupling factors near to 0.2, indicating good interphase between CNF and PVA. Overall, it was found that the use of CNF is clearly advantageous to the use of common cellulosic fibers if superior mechanical properties are desired, but there are still some limitations that are related to processing that restrict the reinforcement content at low contents.

## 1. Introduction

The main objective of a composite, resulting from the combination between two or more materials, is to obtain a new material with unique properties, based on the combination and synergies that were provided by the original ones, as well as reducing production costs and environmental impacts [1,2,3]. In this search, several thermoplastic and thermosetting polymers have been reinforced with a wide variety of fillers, both for technical and economic purposes. However, glass, carbon, or aramid fibers are typical fillers for such thermoplastic materials [4,5]. These composite materials, due to their greater characteristics when compared to the neat matrix, are currently being commercialized and accepted by the market, to the point that they are even considered as commodities (i.e., polypropylene reinforced with 30% of glass fibers). However, although they exhibit excellent mechanical properties, they also present important drawbacks and limitations related to their low recyclability, unhealthy manipulation of the reinforcement, and null sustainability [6]. While it is true that natural fiber reinforced composites are penetrating into the market in the recent years, especially in the form of wood plastic composites (WPC) for the building and automotive sector, their market share is still low when compared to glass or carbon fiber reinforced composites [7,8]. These composites are usually based on polypropylene or polyethylene and reinforced with high-yield fibers, such as stone groundwood (SGW) or thermomechanical pulps (TMP), or even wood sawdust [3,7,9].

In recent years, nanostructured cellulose fibers have been developed with the purpose of obtaining a fully bio-based, biodegradable, and biocompatible material with improved properties when compared to common cellulose fibers [10]. Nanocellulose (NC) presents high specific surface, aspect ratio, transparency, low density (similar to native cellulose), high intrinsic mechanical properties, and a reactive surface due to the presence of hydroxyl groups [11,12,13,14]. As in the case of common cellulosic fibers, they can be obtained either from softwood, hardwood, plants, or even agriculture residues, and by means of different treatments [15,16,17,18,19,20]. NC can be even obtained from bacteria, such is the case of bacterial cellulose (BC). Recently, the Technical Association of Pulp and Paper Industry (TAPPI) proposed a standardization in terms and definitions for cellulose nanomaterials [21]. By definition, cellulose nanomaterials can be divided into two categories: nano-objects and nano-structured, which, at the same time, each category is divided into two subcategories: cellulose nanocrystal (CNC) and cellulose nanofibers (CNF) for nano-objects and cellulose microcrystal (CMC) and cellulose microfibril (CMF) for nano-structured. As expected, the classification is based on morphology and crystallinity degree. Their unique properties confer them the capability to be used in several sectors, such as papermaking, paints, biomedicine, composites, and many other sectors [22,23,24,25,26,27,28]. Despite all of the abovementioned promising applications, the use of NC in thermoplastic composites is still challenging, mainly due to the hydrophobic character of most of the matrices, the water content of NC, and its hydrophilic character. In fact, several works have reported the incorporation of NC into polymer matrices by means of solvent-exchange techniques, as well as dissolving the matrix in order to promote the dispersion of the reinforcement in compatible solvents [29,30].

Among the abovementioned categories, CNF deserve special attention. CNF can be obtained by means of several methods, i.e., oxidation, enzymatic hydrolysis, cationization, or even fully mechanical methods [17,31,32,33,34]. Each treatment, which is usually followed by a fibrillation step that might include high-pressure homogenization, microfluidization, or grinding, leads to significantly different CNF in terms of morphology, properties, characteristics, and, thus, potential applications [16,34,35,36]. Thus, it is well known that TEMPO-mediated oxidation leads to relatively long and thin fibers with a great surface area, while other treatments are based on the reduction of the cellulose chain length, such is the case of enzymatic hydrolysis. In addition, the ability to form structured networks and stable suspensions will depend on the morphology of the CNF, but also on the surface charge thereof [12,37,38]. In addition, CNF present significant advantages to CNC in some applications, such is the case of nanocomposites. The use of CNF as polymer reinforcement can be advantageous to CNC if a strong and entangled network is desired inside the nanocomposites, according to Eichhorn et al. (2010). This mainly comes from the ramifications that can be found in CNF, as well as their ability to bend and greater surface area [25].

Polyvinyl alcohol (PVA) is a well-known polymer that is used for several purposes, such as water-soluble detergent capsules, coating in papermaking, film-forming for packaging, and others [39,40].

The integration of these two phases in one seems to be a good way to form eco-sustainable composites due to the chemical structure of both PVA and natural fibers. The presence of hydroxyl groups in cellulosic fibers, and even some aromatic hydroxyls from lignin in the case of unbleached fibers, glimpse a good interface between polymer matrix and reinforcement. In fact, in the case of unbleached fibers or nanofibers, this interaction between aromatic hydroxyl groups present in lignin has been previously reported for polyolefin-based composites in the presence of maleated coupling agents or even with PVA matrices [3,41,42]. PVA has been widely used for the preparation of natural fiber reinforced composites, containing micro and nanostructured fibers, as well as lignocellulose [39]. However, the water sensitiveness of PVA, together with the high hydrophilicity of natural fibers, seems to jeopardize the dimensional stability of the resulting composites. In fact, Cinelli et al. (2006) found that low temperatures were required for PVA/natural fiber composites processing due to the low thermal stability of cellulosic fibers and, thus, the use of plasticizers was required to avoid excessive pressures in the injection units. In addition, plasticizers were also helpful to decrease the brittleness of the resulting materials, but, at the same time, the tensile strength [43].

There are some works dealing with the incorporation of microfibrillated cellulose (MFC), cellulose nanocrystals (CNC), and cellulose nanofibers (CNF) to PVA matrices. However, none of these works studied the incorporation of these nanostructured cellulosic materials through conventional techniques, such is extrusion, followed by injection molding. For instance, Peresin et al. (2010) prepared electrospun mats containing PVA and CNC (0–15 wt %), but only mechanical improvement on the storage modulus was observed due to the strong interactions between the matrix and the reinforcement [44]. Liu et al. (2013) developed PVA-based nanocomposites that were reinforced with CNF by means of solution casting method, obtaining mechanical properties enhancement significantly low while taking the reinforcing potential and intrinsic properties of CNF into account [45]. More recently, Espinosa et al. (2019) showed the effect of incorporating lignin-containing CNF into PVA matrices, obtaining a significant enhancement of the tensile strength. However, the authors adopted a solvent casting strategy as in many other works, thus limiting the industrial viability of such nanocomposites [42]. Indeed, the interesting properties of CNF against common cellulosic fibers should allow for the addition of low amount of CNF with important effects and improvements on the final properties of the nanocomposites. Other approaches, such is the case of MFC-reinforced PVA nanocomposites, did not show significant advantages to the use of common cellulosic fibers, apart from the benefits on composite production and processing [40].

While there is no doubt that the use of natural fibers in polymer composites as substitutes of those based on glass, aramid, or carbon, is a good approach, when reviewing the available literature, it seems that there are some controversies about the advantages and disadvantages of using nanostructured cellulose fibers as thermoplastic reinforcement [25,46,47]. Concretely, Lee et al. (2014) indicated that more than 30 vol. % of CNF is required to significantly improve the mechanical properties of polylactic acid (PLA), hindering the dispersion of the reinforcement within the matrix unless they are not chemically modified to balance their hydrophilic/hydrophobic character [46]. This issue was also revealed in other works dealing with the use of such nanostructured cellulosic fibers as reinforcement, pointing that one of the biggest issues is to avoid CNF agglomeration [39]. Nonetheless, it must be pointed out that the interface between natural fibers and PLA, even at the microscale, is something still unsolved and requires further research [48,49,50].

The properties of thermoplastic composites result from a combination of the fiber and matrix properties and the ability to transfer stresses across the fiber-matrix interface, i.e., the interfacial shear strength (IFSS), according to Thomason (2002) [51,52]. Among the different parameters presenting significant impact on the final balance of properties of composites, the volume fraction of reinforcement, its dispersion and orientation within the matrix, its morphology, the interaction between fiber and matrix (IFSS), and the intrinsic properties of the reinforcement are of prime importance [53]. When considering a system that is composed by cellulose-based reinforcement and PVA, apparently, the interaction between fiber and matrix can be assumed to be satisfactory, promoting the transfer of stress from matrix to reinforcement. Regarding the dispersion and orientation, it must be considered that it will mainly depend on the compounding and processing strategy. Thus, it is expected that, in the abovementioned works, the distribution of the reinforcement was appropriate, but CNF or CNC were randomly oriented, leading to completely isotropic structures [42,44,54]. On the other side, there are those materials processed by means of conventional techniques, such as extrusion and/or injection molding. Such composite materials usually exhibit a slight orientation of the reinforcement, which is mainly induced by the injection molding process [51,55]. The aspect ratio and specific surface of reinforcement also play a key role in the mechanical performance of the resulting composites [25]. In fact, one of the main advantages of using nanostructured fibers as thermoplastic reinforcement is the elimination of defects or weaker parts of the original fibers that would act as the starting point of cracks. In addition, the increase on the interfibrillar bond densities presumably creates a structure that favors ductility and bending strength, as well as an increase on the strain at break [25,56]. Finally, the intrinsic properties of fibers has been usually determined by means of micromechanical modelling or zero-span tensile tests [51,55,57,58,59,60,61]. However, zero-span tensile tests may lead to error during measuring due to test conditions and/or defects on the tested filaments or fibers and, in addition, it is unconceivable for nanosized reinforcement. On the other hand, micromechanical analysis has been traditionally conducted for microsized fibers and the volume of the interface between the matrix and reinforcement is usually neglected [62].

For all of the above, the present work aims at evaluating the reinforcing potential of CNF prepared by means of TEMPO-mediated oxidation in injection-molded PVA nanocomposites and determining the micromechanical parameters that influence the resulting properties. As reference, SGW was selected mainly due to its commercial availability and its extended use as reinforcement of WPC, as mentioned above. Further, the available literature on the use of SGW as reinforcement is extensive. Indeed, this is a limitation of the present work that should be addressed in future research comparing, for instance, the obtained results with other pulp fibers as PVA reinforcement. In addition, the present work proposes a method for nanocomposites preparation that is based on conventional techniques and analyzes the micromechanical aspects with slight modifications for nanosized reinforcement, obtaining, in the end, the intrinsic tensile properties of CNF.

## 2. Materials and Methods

### 2.1. Materials

SGW pulp was kindly supplied by Zubialde S.A. (Aizarnazabal, Spain) and it was used as reinforcement with no further treatment for comparison purposes. Torraspapel S.A. (Sant Joan les Fonts, Spain) kindly supplied bleached kraft pine fibers (BKPF), from Celulosa Arauco y Constitución (Constitución, Chile), and they were used for the production of CNF and then used as reinforcement. PVA (*M*_w_ 30,000–70,000, 87%–90% hydrolyzed) was used as polymer matrix. All of the required reagents for the characterization and CNF production were supplied by Sigma–Aldrich (Barcelona Spain) and they were used as received.

### 2.2. Preparation of CNF

TEMPO-mediated oxidation was performed maintaining pH at 10, according to the methodology that was reported by Isogai et al. (2011) [11]. In a typical experiment, 15 g of BKPF were suspended in 1500 mL of water containing dissolved NaBr and TEMPO (2,2,6,6-tetramethylpiperidine-1-oxyl radical). The amount of NaBr and TEMPO was calculated to be 100 and 16 mg/g of BKPF, respectively. The suspension was kept under gentle stirring for 15 min. in an IKA RW28 Basic stirrer to assure a good dispersion of all the substances. Subsequently, 8 vol. % NaClO solution was added drop-wise to the slurry, maintaining the pH at 10. The volume was calculated to add 5 mmol of NaClO per g of BKPF. Afterwards, a 0.1 M NaOH solution was gradually added until no significant changes on pH were observed. The oxidized suspension was then washed with distilled water until obtaining a neutral pH in order to remove any trace of non-reacted reagents and catalyst.

Finally, the trated suspension (2 wt % consistency) was gradually passed through a high-pressure homogenizer (PANDA Plus 2000, Gea Niro Soavi, Parma, Italy) operating at different pressures. The selected sequence was three passes at 300-bar, three passes at 600-bar, and three passes at 900-bar, as previously reported [28]. The pressure was gradually increased to avoid clogging in the pressure chambers. The resulting gel-like suspension was stored in plastic bottles and preserved at 4 °C for further use and characterization.

### 2.3. Characterization of the Reinforcement

#### 2.3.1. Characterization of CNF

Carboxyl content (*CC*) was measured prior to high-pressure homogenization in order to determine the effectiveness of the oxidative treatment. This determination was conducted by means of conductimetric titration in a Meter Basic 30 (Crison Instruments, S.A., Alella, Spain). For this, 100 mg of oxidized BKPF were suspended in 15 mL of a 0.01 N HCl solution and kept under gentle stirring for 10 min. This process might exchange the Na^+^ cations from the COO^−^ groups at the cellulose surface by protons H^+^. The suspension was then titrated with a 0.01 M NaOH solution, recording the conductivity of the suspension every 0.1 mL addition. The titration curve exhibited the presence of a strong acid (excess of HCl) and a weak acid (carboxylic acid). The carboxyl content was then calculated according to Equation (1).

(1) $$CC=162\cdot c\cdot \left(V_{2}-V_{1}\right)\cdot \left(w-36\cdot \left(V_{2}-V_{1}\right)\right)$$

where *V*_1_ and *V*_2_ are the equivalent volumes of the added NaOH in liters, c is the concentration of the NaOH (0.01 M), and *w* is the dry weight of the sample in grams.

Colloidal titration determined cationic demand (*CD*) using a particle charge detector Mütek PCD04 (BTG GmbH, Weßling, Germany). This titration was performed through the surface adsorption of polydiallyldimethylammonium (polyDADMAC) and the excess was titrated with poly(ethylene sulfonate) sodium salt (Pes-Na), both from BTG (Weßling, Germany). The molecular weight (*M*_W_) of the polyDADMAC was 107 kDa. First of all, a CNF suspension at 0.04 wt % consistency was prepared and dispersed while using an Ultraturrax T25 (IKA, Königswinter, Germany) at 20,000 rpm for 10 min. From this suspension, a 10 mL-aliquot was taken and then mixed with 25 mL of polyDADMAC for 5 min. under gentle stirring. The suspension was then centrifuged for 90 min. at 1254 G-force in a Sigma Laborzentrifugen (6K15). Then, 10 mL of the supernatant were taken to the particle charge detector and titrated with 0.01 N Pes-Na until the display exhibited a potential of 0 mV. The *CD* was finally calculated according to Equation (2):

(2) $$CD=\frac{\left(C_{polyDADMAC}\cdot V_{polyDADMAC}\right)-\left(C_{Pes-Na}\cdot V_{Pes-Na}\right)}{w_{sample}}$$

where *C_polyDADMAC_* and *V_Pes-Na_* are the concentration and volume of the polyDADMAC, respectively, *C_Pes-Na_* and *V_Pes-Na_* are the concentration and volume of Pes-Na, respectively, and *w_sample_* is the dry weight of sample.

The degree of polymerization (DP) was determined by means of viscosimetric measurements of dissolved cellulose in cupriethylenediamine, according to UNE 57-039-92 and Henriksson et al. (2007) [17]. Briefly, the viscosimetric average molecular weight was calculated from Equation (3), where *η* is the intrinsic viscosity, *K* equals to 2.28, and *a* equals to 0.76. The relationship between the intrinsic viscosity and the degree of polymerization can be described by Mark-Howink-Sakurada equations.

(3) $$\eta =KM^{a}$$

The specific surface area was estimated from the carboxyl content and cationic demand, as previously reported [10]. For this, it was considered that the interaction between CNF surface and the added polyDADMAC during *CD* determination occurred through two mechanisms. On the one hand, interaction between carboxyl groups and polyDADMAC and, on the other, surface adsorption. In addition, it was assumed that the surface adsorption of polyDADMAC took place as a monolayer and that polyDADMAC exhibits a cylindrical geometry. The first assumption, related to the monolayer adsorption, can be considered due to the molecular weight of the used polyDADMAC, as previously reported by the authors [63]. Thus, the surface of polyDADMAC was estimated by calculating the monomer’s surface and its degree of polymerization (667), while also considering the bond distances. These dimensions were previously calculated by Espinosa et al. (2016) and they accounted for 5.427 and 4.849 Å for diameter and length, respectively. Thus, the theoretical CNF’s surface area can be calculated through Equation (4).

(4) $$\sigma_{CNF}=\left(CD-CC\right)\cdot \sigma_{p+}$$

where *σ_CNF_* and *σ_P+_* are the specific surface areas of CNF and polyDADMAC, respectively. The diameter of CNF can be calculated thus assuming that CNF exhibited a cylindrical geometry. The length of CNF was estimated according to a previously reported methodology, which was based on the DP [64].

Transmission electron microscopy (TEM, Carl Zeiss Vision, Madrid, Spain) was performed on a diluted suspension of CNF in the presence of uranyl acetate. The images were taken in a Zeiss EM910 with a resolution of 0.3 nm.

#### 2.3.2. Characterization of SGW

Morphological analysis was carried out using a MorFi Compact analyzer (TechPap, Grenoble, France), which is able to calculate, among other parameters, the average length and diameter. This equipment analyzes an average of 30,000 fibers per test and it is equipped with a CCD camera for this purpose.

### 2.4. Composites Preparation

For comparison purposes, composite materials containing 20, 30, and 40 wt % of SGW were prepared. On the other hand, nanocomposites containing 2.25, 3.375, and 4.50 wt % of CNF were prepared. In all cases, the PVA matrix and reinforcement were mixed in a Brabender blender at 80 rpm for 20 min. The temperature was set at 190 °C for SGW composites and at 210 °C for CNF composites. For comparison purposes, the neat matrix was also processed in the Brabender blender prior to injection molding. The resulting materials were then cooled down and milled in a knife mill equipped with a 1 mm sieve at the bottom. The resulting composites were then processed using an injection molding Meteor 40 (Mateu & Solé, Barcelona, Spain) equipment operating at 220 °C in the three areas. The first and second injection pressures, measured at the hydraulic system, were set at 12.25 and 4.90 MPa, respectively. The tensile specimens were fabricated according to the ASTM D638 standard.

### 2.5. Characterization of the Composites

The tensile test specimens were conditioned in a climate chamber at 23 °C and 50% of relative humidity for 48 h prior mechanical testing (ASTM D638). The specimens were then tested according to the ISO 527 standard using an Instron 1122 Universal Testing Machine (Metrotec S.A., Lezo, Spain) that was equipped with a 5 kN load cell and two gauges of 35 mm in width and 60 mm in length, and operating at a rate of 5 mm/min. The Young’s modulus was determined by means of an axial extensometer. A minimum of ten specimens were tested in each case.

Cross-sectional observation on the broken surfaces of the CNF-reinforced composites was performed by means of field emission scanning electron microscopy (FE-SEM, Carl Zeiss Vision, Madrid, Spain). For this, a Zeiss DSM 960 microscope was used and the samples were bound to the metal holder while using carbon tape and coated with a thin layer of gold.

### 2.6. Micromechanical Analysis

The micromechanics of the SGW reinforced PVA composites was carried out using the modified Kelly and Tyson equation [65]. The equation was solved for the composite containing 40 wt % of SGW, following the methodology that was proposed by Bowyer and Bader [2]. The proposed methods were applied according to the literature [2,55,65,66]. Once solved, the equation returns the values of the interfacial shear strength (*τ*), the orientation factor (*χ*_1_), and the intrinsic tensile strength of SGW as PVA reinforcement (*σ_t_^F^*). The interfacial shear strength and the orientation factor were used as input for the Kelly and Tyson modified equation in order to obtain a theoretical intrinsic tensile strength of the SGW.

The micromechanics of nanocomposites have some particularities with respect to short fiber-reinforced composites. The main concern is related to the thickness of the interface between the matrix and the reinforcement. In the case of microsized reinforcement, the thickness of such interface can be neglected, as the ratio between the interface thickness and the fiber radius is low. In the case of nanofibers, the thickness of the interface cannot be neglected by micromechanics models [67,68,69].

The Kelly and Tyson modified equation has been widely used to evaluate the micromechanics of the tensile strength of short fiber reinforced composites [55,65,70,71]. The original model is based on an interface with negligible thickness, but can be adapted to the case of nanocomposites. In this sense, Zare proposed a combination of Kelly-Tyson and Pukanszky models as [62,72]:

(5) $$\sigma_{t}^{R}=\frac{\chi_{1}\cdot \alpha \cdot \tau \cdot V^{F}}{\sigma_{t}^{m}}+1-V^{NF}$$

where *σ_t_^R^* is the relative yield strength of the nanocomposite as the ratio between the tensile strengths of the composite (*σ_t_^NC^*) and the matrix (*σ_t_^m^*). The orientation factor (*χ*_1_) equalizes the effect of the orientation of the fibers against the loads. This parameter is known to be mainly impacted by the equipment and the geometry of the injection mold used to manufacture the specimens. Orientation factor equals 1 for totally oriented fibers, 3/8 for two-dimensional (2D) random fibers and 1/5 for three-dimensional (3D) random fiber configurations [73]. The factor α refers to the aspect ratio of the nanofibers (ratio between its mean length (*L^NF^*) and diameter (*d^NF^*)). The interfacial shear strength (*τ*) measures the capacity of the interface to transmit shear loads from the matrix to the reinforcements. Finally, *V^NF^* is the nanofiber content as volume fraction.

Zare proposed the inclusion of the interface thickness (*t*) in the equation:

(6) σtNC=χ1·α·τ(1+tdNF2)·VNF+σtm[1−(1+tdNF2)2·VNF]

Equation (6) presents three unknowns, the orientation factor, the interface thickness and the interfacial shear strength. The authors used a 0.33 value based on the literature [74]. This value is higher than 1/5 because the nanofibers are expected to show semi orientated arrangements like in the case of microfibers [71,75]. Subsequently, the interfacial shear strength can be obtained from:

(7) $$\tau =\frac{\sigma_{t}^{m}\cdot \left(B-2.04\right)}{\chi_{1}\cdot \alpha }$$

With that value, Equation (7) can be used to compute the interface thickness, and the interface strength (*σ_t_^I^*) can be obtained from:

(8) σtI=σtm·e(B1+2·tdF2)

With

(9) $$B=\frac{Ln\;\left(\sigma_{t}^{R}\cdot \frac{1+2.5\cdot V^{NF}}{1-V^{NF}}\right)}{V^{NF}}$$

In contract with the original Kelly and Tyson equation, the modified model does not allow for approximating the value of the intrinsic tensile strength of the nanofibers. Anyhow, if the mean length of the nanofibers is considered to be lower that the critical length (*L_c_*), the use of the definition of a critical length can be used to establish a lower bound for the intrinsic tensile strength [66]:

(10) $$L^{NF}\leq L_{C}=\frac{\sigma_{t}^{F}\cdot d^{NF}}{2\cdot \tau }\underset{}{\Rightarrow }\sigma_{t}^{NF}\geq \frac{L^{NF}\cdot d^{NF}}{2\cdot \tau }$$

When microsized fibers are used for micromechanical modelling, all of the subscripts containing “*NF*” should be changed to “*F*”.

## 3. Results and Discussion

### 3.1. Characterization of the Reinforcement

Prior to composites preparation, CNF were characterized, as described in the previous section. CNF exhibited a cationic demand (CD) of 1238 µeq/g and the carboxyl content (CC) accounted for 793 µeq/g. When considering the amount of NaClO that was used as oxidizing agent, the obtained values for CD and CC are in consonance with previously reported results [10,28]. In addition, the obtained diameter and specific surface area, derived from the calculations detailed above, indicated that the obtained cellulose nanomaterial can be classified as CNF. Concretely, the diameter accounted for 12.3 nm and the specific surface area, for 216.72 m^2^/g. This specific surface area indicates that the presence of free hydroxyl groups at the surface of the CNF is significantly high, glimpsing that the ability to bond PVA will be notable by means of hydrogen bonding due to its chemical structure. The degree of polymerization (DP) gives an indication of the length of the CNF, as previously described by Shinoda et al. (2012). In fact, the authors reported a correlation between the DP and the length of CNF [64]. The length could be estimated to 1.53 µm if the same relationship is applied to the obtained results, and when considering that CNF exhibited a DP of 534. Thus, the aspect or slenderness ratio of the obtained CNF accounted for 124.4. The aspect ratio of reinforcement has been reported to be one of the main parameters influencing the strength of composites, where high values are pursued. However, the aspect ratio usually accounts for significantly lower values in the case of microsized fibers [76,77]. In fact, this is one of the main reasons why CNF-reinforced composites show higher mechanical properties than fiber-reinforced composites [46,78,79]. Comparatively, SGW exhibited a length and diameter of 643 and 26.9 µm, respectively, resulting in an aspect ratio of 23.9. In fact, this aspect ratio should allow for SGW to impart significant improvements on the PVA matrix, since it is widely accepted that fibers with aspect ratios that are higher than 10 may contribute to reinforce the polymer matrix [77]. However, in the case of CNF, some authors have reported that at least an aspect ratio of 50 is required to glimpse any reinforcing effect [25,80].

**Figure 1:**
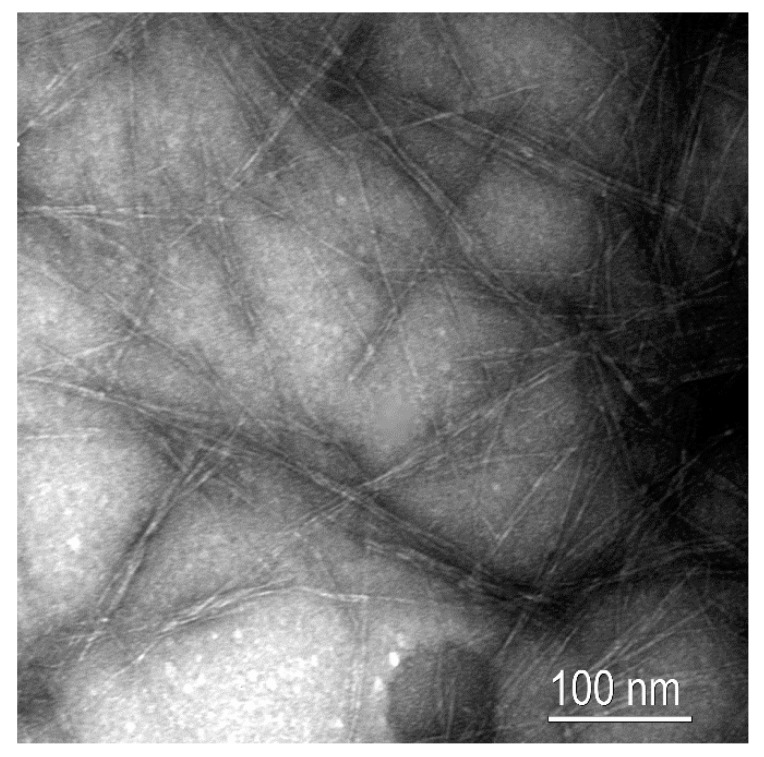
Transmission electron microscopy (TEM) image of the obtained cellulose nanofibers (CNF).

Figure 1 shows a TEM image of the CNF suspension, where the high aspect ratio can be observed, as well as the diameters of the same magnitude than those calculated above. In addition, although the CNF suspension was significantly diluted, different contact points can be observed among the different nanofibers. This fact reveals the ability of CNF to form entangled networks that may confer ductility and strength to the resulting nanocomposites, as will be later discussed.

### 3.2. Macromechanical Evaluation of the Composites

**Figure 2:**
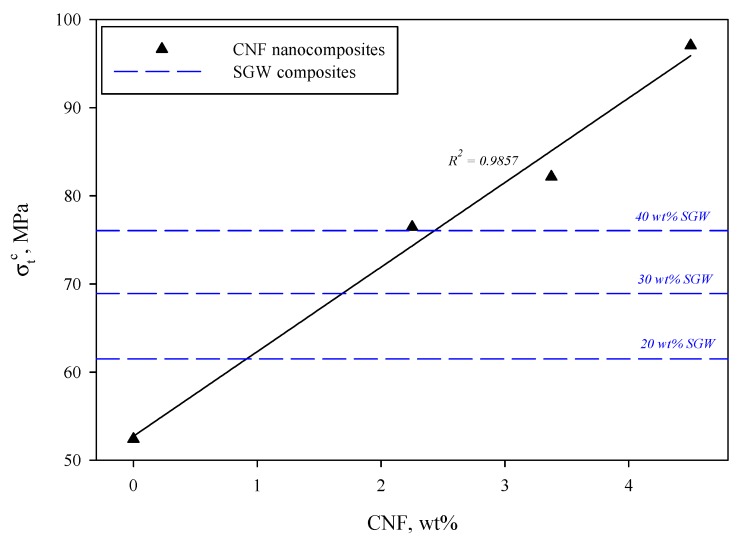
Evolution of tensile strength (*σ_t_^c^*) as the amount of CNF was increased. Blue dashed lines indicate the tensile strength of polyvinyl alcohol/stone groundwood (PVA/SGW) composites at different SGW content for comparison purposes.

Moderate amounts of CNF were incorporated into the PVA matrix, ranging from 2.25 to 4.50 wt %, as explained above. The amount of CNF was limited to 4.50 wt % due to processing restrictions, since the amount of water that CNF retain is high when compared to microsized fibers. The neat PVA matrix exhibited a tensile strength of 52.40 MPa, while the Young’s modulus accounted for 5.10 GPa. This strength is significantly higher than most of the polyolefin commodities, such as polypropylene (PP) or polyethylene (PE), and of the same magnitude than polylactic acid (PLA), according to previously published works [3,48,81]. Regarding the Young’s modulus, it is clear that PVA is stiffer than the aforementioned materials [41,82]. The evolution of the tensile strength followed a linear regression with a correlation factor *R*^2^ = 0.9857 (Figure 2), indicating that both dispersion and interface were successfully achieved. In fact, this linear behavior fits the rule of mixtures, which is widely used to describe the mechanical performance of composite materials. For comparison purposes, Figure 2 also shows the evolution of tensile strength when different percentages of SGW, ranging from 20 to 40 wt %, were incorporated into the PVA matrix.

The incorporation of 2.25 wt % CNF increased the tensile strength in a 45.9% and an improvement of 85.2% was achieved at the maximum dosage of CNF, i.e., 4.50 wt %. This great improvement with such low reinforcement content can be only achieved if the intrinsic tensile properties of the reinforcement are extremely high. In fact, the incorporation of 40 wt % of SGW into the PVA matrix resulted in a similar tensile strength than when 2.25 wt % of CNF were used as reinforcement. The linear tendency can be attributed to the good dispersion of the CNF within the PVA matrix, as well as the good interface that the system presents. The amount of hydroxyl groups at the CNF surface is expected to be high, which is mainly due to the huge specific surface that they present. Thus, the interactions with the PVA matrix will be presumably abundant and based on hydrogen bonding, promoting the stress transfer from the matrix to the reinforcement when the nanocomposite is subjected to stress [39,70,83]. CNF generate an expanded high-volume and interconnected web-like structure within the matrix that inhibits the crystallization of PVA, suppressing the free movement of polymer chains and promoting the generation of multiple bonds between polymer and reinforcement, as reported by Nakagaito et al. (2005). With convenient fabrication method, the nanocomposites made of PVA/CNF could replace traditional non-biodegradable plastic and composite materials in many applications, including the automotive sector or even ski poles [40,84]. Although Figure 2 does not show the evolution of PVA/SGW, the evolution of the tensile strength of such composites also followed a linear tendency. In fact, although SGW contains a significant amount of lignin that could hinder the interaction with PVA, in previous works it has been demonstrated that this type of fiber also interacts by means of hydrogen bonding with other matrices, such is the case of maleated polypropylene. Thus, it has been extensively reported that a linear tendency as the amount of reinforcement is increased denotes good interaction between both phases, which means that synergy exists between the properties of the reinforcement and the matrix as function of the ratios inside the composite [41,48].

Comparatively, the obtained nanocomposites exhibit superior mechanical properties than other PVA/CNF attempts that are available in the literature. For instance, Espinosa et al. (2019) did not report any significant improvement on tensile strength when the content of TEMPO-oxidized lignocellulosic nanofibers was increased from 1 to 7 wt %, indicating that their dispersion was probably not assured. In addition, the improvement was quantified in 46.7% in almost all cases, while this improvement was achieved with a 2.25 wt % load of CNF in the present work, being significantly improved at higher dosages. However, in that case, the selected CNF contained lignin, which might hinder the interaction with PVA [42]. Liu et al. (2013) also reported a significant improvement on tensile strength due to the incorporation of CNF, obtaining a relative improvement of 19.8% for a 3 wt % CNF dosage. Nonetheless, the authors incorporated high amounts of CNF into the PVA, reaching reinforcement loads of 60 wt %. In addition, the neat PVA that Liu et al. (2013) proposed exhibited a tensile strength of 29.7 MPa, being considerably lower than the selected matrix in this work [45]. In any case, there is no doubt that CNF, and even microfibrillated cellulose, have great potential as reinforcement of PVA, as broadly discussed by Tan et al. (2015) in their review [39].

The great reinforcing potential of CNF can be attributed to a combination of different factors. Apart from the interactions between reinforcement and matrix, the aspect ratio of the reinforcement is also a parameter of prime importance, as described by Thomason (2002) [51]. The obtained CNF exhibited an aspect ratio of 124.4, while, in the case of SGW, this ratio accounted for 23.9, as described above. Another parameter that deserves special mention is the intrinsic tensile strength of the CNF, as will be later discussed. Alcalà et al. (2013) reported that the intrinsic tensile strength of TEMPO-oxidized CNF could be quantified in almost 7 GPa, value significantly higher to that reported for common cellulose fibers, which might range from 0.14 to 2 GPa, depending on the origin and type of fiber [41,55,58,85,86].

Lee et al. (2014), in their review, reported that at least 30 wt % of CNF as reinforcement is required to obtain significant improvements with respect to the matrix, which was PLLA [46]. In that case, the interface between reinforcement and matrix was not assured due to the presence of methyl groups on PLLA chain. Contrarily, the proposed matrix in this work assures the obtaining of a well-bonded system, showing that low amounts of CNF are required in order to obtain similar results than those obtained with regular microsized fibers. Qiu and Netravali (2012) studied a similar approach, where PVA/MFC composites were produced by means of extrusion and solution casting. In that work, the authors prepared composites with MFC ratios from 5% to 50% by weight and, in some cases, glyoxal crosslinking was required. However, the intrinsic properties of the microfibrillated cellulose may be significantly lower, as well as aspect ratio, thus the achieved enhancements were much lower than the ones obtained in this work [40].

**Figure 3:**
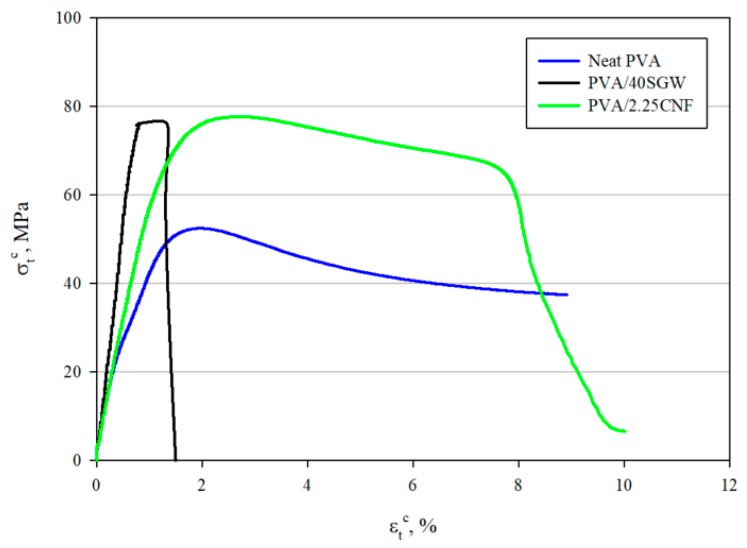
Stress-strain curves of neat PVA and PVA/2.25CNF and PVA/40SGW composites.

Although the obtained tensile strength of the nanocomposite reinforced with 2.25 wt % was of the same magnitude than in the case of reinforcing PVA with a 40 wt % of SGW, their behavior under stress was completely different. In fact, it has been reported that low amounts of CNF, apart from increasing the tensile strength and modulus, also contribute to the toughness of the resulting material. Figure 3 shows the stress–strain curves of neat PVA, the nanocomposite reinforced with 2.25 wt % of CNF and, for comparison purposes, the PVA composite reinforced with 40 wt % of SGW.

The stress–strain curves show the behavior of the tested materials. PVA shows an initial elastic region with a soft transition to the plastic one, indicating a typical shape for ductile materials. The yield and ultimate strengths are similar and not far in terms of strains, but the fracture strength was impossible to measure. On the other hand, the SGW reinforced PVA composite showed a brittle behavior, with a clear elastic region and a noticeable decrease of the plastic region when compared with the matrix. The yield, ultimate, and fracture strengths are very similar and not distant in terms of strain. CNF reinforced PVA nanocomposites showed a ductile behavior like the matrix, possibly due to the low nanofiber content, being unable to change the fracture mechanism from ductile to brittle, but increasing the tensile strength and due to the presumably three-dimensional (3D) network that they are able to conform within the matrix. In the case of the nanocomposites, there is an elastic region, with a slope between the matrix and the SGW-based composite. The yield strength of the nanocomposite seemed to be noticeably lower than its ultimate strength. In fact, the yield strength of the SGW-based composite is higher than the nanocomposite. The ultimate strength is at the end of a soft curve after a low deformation of the specimen. Further deformations of the nanocomposite specimen decreased its area and its strengths up to its strength at fracture, being noticeably far from its ultimate strength.

In the case of neat PVA, toughness was estimated to be higher than 4.07 MJ/m^3^, since the test was stopped before breaking. In the case of the PVA/2.25CNF, toughness accounted for 5.32 MJ/m^3^, indicating that, as previously reported, the incorporation of long and slender nanosized fibers also benefits this property [39,87]. Contrarily, the incorporation of microsized fibers, such is the case of SGW, significantly decreased this parameter to 0.71 MJ/m^3^. The strain at break, another parameter that provides information regarding the behavior of materials, was significantly different. Overall, the obtained toughness in the PVA/2.25CNF was higher than values that were reported for PP, PLA, HDPE, or even collagen, while the tensile strength was significantly superior [48,53,88]. Nevertheless, the obtained values were lower than those that were reported for natural rubber, CNF/HEC (hydroxyethyl cellulose) composites, and nanopapers, among others [87].

**Figure 4:**
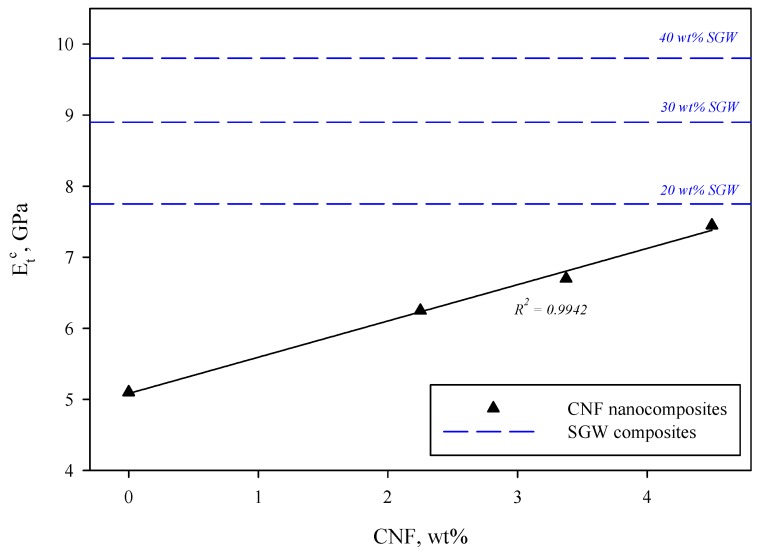
Evolution of Young’s modulus (*E_t_^c^*) as the amount of CNF was increased. Blue dashed lines indicate the Young’s modulus of PVA/SGW composites at different SGW content for comparison purposes.

When considering the elastic regions of the stress-strain curves, it becomes apparent that, when PVA was reinforced, regardless the type of reinforcement, Young’s modulus was increased. The effect of CNF resulted in a lower impact than the incorporation of SGW, as can be corroborated in Figure 4. This fact can be surprising, especially when taking into account that CNF have been reported to be stiffer than regular lignocellulosic fibers [58,85]. The incorporation of 4.50 wt % of CNF into the PVA matrix increased the Young modulus to 7.45 GPa, value of the same magnitude than that obtained with 20 wt % of SGW. However, the incorporation of such an amount of CNF increased the tensile strength to 97.05 MPa, an unconceivable value if SGW fibers were used. CNF have been reported to significantly increase the Young’s modulus of PVA, reaching enhancements of 210% when 15 wt % CNF loads are used [39,45]. For TEMPO-oxidized nanofibers, Espinosa et al. (2019) also found an improvement of almost 100% when 7 wt % of CNF were incorporated into the PVA matrix [42].

**Figure 5:**
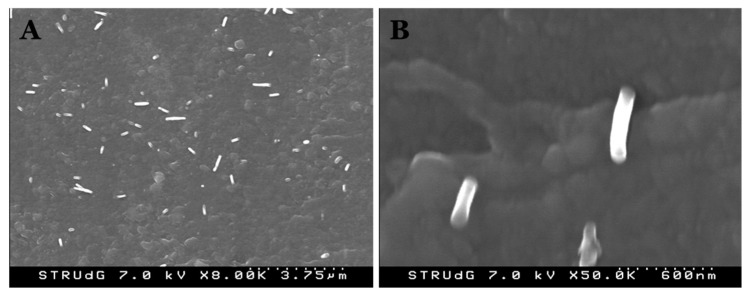
Field emission scanning electron microscopy (FE-SEM) images of the fracture surface at low (**A**) and high (**B**) magnification.

The linear evolution of Young’s modulus as the amount of CNF was increased in the nanocomposites is also an indicator that reinforcement was properly dispersed within the PVA matrix [48,76]. Indeed, this dispersion was clearly observed by means of field emission scanning electron microscopy (FE-SEM), as reflected in Figure 5.

Several nanosized fibrils can be observed at the fracture surface after mechanical testing. Apparently, CNF were properly dispersed in the nanocomposite and no voids were observed, which indicated that no slippage occurred when the nanocomposite was submitted to stress. In addition, although the observed diameters seem to be slightly higher than those calculated above, it might be possible that some bundles were generated during compounding.

### 3.3. Micromechanal Analysis of the Interface of the Nanocomposites

The modified Kelly and Tyson equation was solved for the composite containing 40 wt % of SGW, as explained above. The obtained orientation factor was 0.307. This value is well within the expected 0.25 to 0.35 range, typical from natural fiber reinforced composites processed by means of injection molding in the same facilities than in the case of the present study [66]. In the case of microfiber reinforced composites, von Mises (*σ_t_^m^*/3^1/2^) and Tresca (*σ_t_^m^*/2) criteria are used to obtain a range of values that define strong interfaces. In the case of PVA, the expected values will vary from 30.2 to 26.6 MPa. The interfacial shear strength for PVA/SGW composites was estimated at 26.34 MPa. This value almost fits the Tresca and von Mises criteria, evidencing the presence of a strong interface. In a previous work, where the SGW reinforced polypropylene (PP) composites were prepared and characterized, the interfacial shear strength accounted for 3.6 and 15.8 MPa for uncoupled and coupled materials, respectively, evidencing the importance of providing a good interface between matrix and reinforcement [58]. The value for coupled composites that was reported by López et al. (2011) was significantly low as compared to the interfacial shear strength that was obtained in the present study for PVA-based composites. However, it must be considered that the tensile strength of the matrix limits this parameter. Thus, the obtained differences are understandable when considering that the tensile strength of PP is significantly lower than PVA’s. The same reinforcement exhibited an interfacial shear strength of 19.3 MPa when polyamide 11 (PA11) was used as matrix, which was a value near to Tresca criteria for PA11-based composites [89]. Thus, the PVA-based composites showed interfaces that were weaker than PP-based materials but stronger than PA11 ones. The intrinsic tensile strength of SGW as PVA reinforcement was estimated at 428 MPa. This value is in the range 280 and 618 MPa, being computed for SGW-reinforced PP composites [58]. The literature shows a relation between the chemical nature of the matrix and the intrinsic tensile strength of the reinforcements [90]. The intrinsic tensile strength of a reinforcement that was obtained by using micromechanics is related with the exploitation of the strengthening abilities of the fibers instead of its tensile properties as single fibers. In this sense, SGW-reinforced PA11 composites delivered a value of 562 MPa [89,91].

The obtained orientation factor and interfacial shear strength were used as input for the Kelly and Tyson modified equation with the data for the composites with 20 and 30 wt % SGW contents, thus obtaining theoretical values for the intrinsic tensile strength of the SGW. The obtained values were 401 and 361 MPa, respectively. These values differ from the one that was obtained for the 40 wt % reinforced PVA/SGW composite, but it can be due to the shape of the stress-strain curves of the PVA/SGW composites. PVA/SGW composites show a linear elastic region up to almost the maximum tensile strength (Figure 3). The shape of such curves hinders the application of Bowyer and Bader’s solution to the Kelly and Tyson model. Other composites do not show this region of low slope [58,89]. It was impossible to solve the equation for the composites with SGW contents lower than 40 wt %. Besides, the mean length of the fibers decreases with increasing reinforcement contents and, consequently, the composites with low reinforcement loads will include longer fibers [53]. Thus, it is possible to obtain higher strengthening yields for the same reinforcements with lower intrinsic tensile strength.

The micromechanics study of the PVA/SGW composites showed materials with strong interfaces and a mean exploitation of the strengthening capabilities of SGW.

Following, the micromechanics of the interface of the nanocomposites was examined. Interfacial shear strength (*τ*), interface thickness (*t*), and interface strength (*σ_t_^I^*) computed with Equations (7), (6), and (8), respectively, are shown in Table 1.

The values of the interfacial shear strength are in the range between 31.25 and 25.28 MPa. Thus, the interface can be considered to be quite strong. Other researchers obtained similar strong interfaces. Chopra et al. (2017) obtained an interfacial shear strength around 22 MPa for carbon nanotube reinforced polybutylene terephthalate nanocomposites [73]. The same authors also observed a tendency of the interfacial shear strength to decrease with increasing percentages of reinforcement. This can be related with the increasing difficultly in obtaining good reinforcement dispersions when its percentage is increased. In any case, the values suggest the existence of a relevant interface between the CNF and the matrix.

The obtained values for the thickness of the interface layer are significant when compared to the nanofibers diameter. The theoretical diameter of the interface layer is between 19.6 and 15.2 times higher than the fiber diameter. On the other hand, as compared with the diameter of a microfiber, the diameter of such fiber is between 111.5 and 144.0 times larger than the interface layer thickness. The obtained values are higher than expected and, in some sense, contradict some authors that preview interface thickness always lower than the nanofiber diameter [92]. Chopra et al. (2017) obtained a similar interface layer thickness of such a layer for nanocomposites, and agreed in the fact that the model overestimates the thickness of such layer. The same authors proposed a second equation, based on a modification of combined Leidnar–Woodhams and Pukanszky models for cylindrical nanofillers that returns layer thickness 10 orders of magnitude smaller [73]. However, even if the interface thickness was 10 times smaller, it cannot be considered to be irrelevant in the case of nanocomposites, as usually is in the case of microsized fiber reinforced composites. The values suggest the existence of a wide interface layer around the nanofibers. Such wide interface areas increase the area that is susceptible to matrix nanofiber interactions and, thus, the creation of strong interfaces. Chopra et al. (2017) related the creation of such wide areas with good fiber dispersion and low fiber aggregates creation [73].

The strength of the interface was found to be in the range between 94.8 and 104.2 MPa, clearly higher than the tensile strength of the matrix. The thickness of the interface layer affects the strength of such a layer and, the larger the thickness, the lower the strengths. This was observed by other researchers, who stated that thin interface layers will be always stronger than thick one’s for similar interfacial shear strength values [62,73].

Using the definition for the critical length of a fiber (Equation 10), it is possible to establish a lower bound for the intrinsic tensile strength of the fibers (*σ_t_^F^*) or nanofibers (*σ_t_^NF^*). Table 1 shows the obtained values for the CNF. This values are in the range from 6297.5 to 7622.5 MPa. These values are 15 to 18 times higher than the SGW fibers as PVA reinforcement, showing the notable strengthening abilities of nanofibers in front of microsized fibers. The intrinsic tensile strength of CNF is around nine to 18 times the typical intrinsic tensile strength of a natural fiber [55,66,93]. Moreover, the theoretical intrinsic tensile strength of the CNF will be equal or higher than the obtained values (Equation (10)). This value can then be used to solve a modified rule of mixtures for the tensile strength:

(11) $$\sigma_{t}^{C}=\sigma_{t}^{NF}\cdot f_{c}\cdot V^{NF}+\sigma_{t}^{m}\cdot \left(1-V^{NF}\right)$$

where *f_c_* is a coupling factor that accounts for the impact of the quality of the interface, the mean orientation of the fibers, and the morphology of such fibers. The literature shows that in the case of short fiber semi aligned reinforced composites the coupling factor takes values around 0.2 when a very good interface is obtained [94]. Table 1 shows the obtained coupling factors. All of the values are near 0.2, corroborating the strength of the interface between CNF and PVA.

## 4. Conclusions

In this work, biodegradable PVA nanocomposites reinforced with CNF have been prepared and tested. The matrix was intentionally selected due to its affinity with cellulose, bringing to the light the impact of using CNF instead of microsized fibers. It was found that the incorporation of low amounts of CNF lead to nanocomposites with significantly high tensile strength and toughness. Concretely, the incorporation of a 2.25 wt % of CNF enhanced the tensile strength of the PVA to the same magnitude than when 40% of microsized fibers was added. Further, the incorporation of 4.50 wt % of CNF increased the tensile strength of PVA in an 85.2%, almost reaching 100 MPa of tensile strength. Moreover, a high strain at break was found for the nanocomposites, with no significant reduction with regard to the neat matrix. In addition, the incorporation of CNF into the PVA matrix lead to tougher structures, clearly indicating that a 3D structured network was created within the matrix mainly due to the high aspect ratio of CNF. Regarding Young modulus, the results indicated that the use of nanostructured reinforcement affects, to a lesser extent, the rigidity of the material, corroborating the obtained values for toughness. The linear behavior of Young’s modulus as the amount of CNF was increased indicated that CNF dispersion was properly achieved. This was corroborated by means of FE-SEM observation. From the study of the interface, coupling factors that were near to 0.2 were obtained, which indicated that a good interface was obtained. In addition, the obtained intrinsic tensile strength of CNF was found to be 15 to 18 times higher than SGW in the same matrix. Overall, it was found that the use of CNF is clearly advantageous to the use of common cellulosic fibers if superior mechanical properties are desired, but there are still some limitations that are to processing that restrict the reinforcement content at low contents.

## Figures and Tables

**Table 1:** Micromechanics of the tensile strength of PVA/CNF nanocomposites.

CNF % w/w	*V^NF^* v/v	*τ* (MPa)	*t* (nm)	*σ_t_^I^* (MPa)	*σ_t_^NF^* (MPa)	*f_c_*
2.250	0.0185	31.25	120.6	94.83	7622.5	0.21
3.375	0.0280	27.40	93.4	104.23	6825.6	0.19
4.500	0.0370	25.28	96.2	97.56	6297.5	0.19

## References

[1] George M., Chae M., Bressler D.C. (2016). Composite materials with bast fibres: Structural, technical, and environmental properties. Prog. Mater. Sci..

[2] Bowyer W.H., Bader M.G. (1972). On the re-inforcement of thermoplastics by imperfectly aligned discontinuous fibres. J. Mater. Sci..

[3] López J.P., Mendez J.A., Espinach F.X., Julian F., Mutjé P., Vilaseca F. (2012). Tensile strength characteristics of polypropylene composites reinforced with stone groundwood fibres from softwood. Bioresources.

[4] La Mantia F.P., Morreale M. (2011). Green composites: A brief review. Compos. Part A Appl. Sci. Manuf..

[5] Seal A., Bose N.R., Dalui S.K., Mukhopadhyay A.K., Phani K.K., Maiti H.S. (2001). Mechanical properties of glass polymer multilayer composite. Bull. Mater. Sci..

[6] Greenberg M.I., Waksman J., Curtis J. (2007). Silicosis: A Review. Dis. Mon..

[7] Najafi S.K., Hamidinia E., Tajvidi M. (2006). Mechanical properties of composites from sawdust and recycled plastics. J. Appl. Polym. Sci..

[8] Dwivedi D. (2017). Wood Plastic Composites Market by Type—Global Opportunity Analysis and Industry Forecast, 2017–2023.

[9] Tarrés Q., Melbø J.K., Delgado-Aguilar M., Espinach F.X., Mutjé P., Chinga-Carrasco G. (2018). Bio-polyethylene reinforced with thermomechanical pulp fibers: Mechanical and micromechanical characterization and its application in 3D-printing by fused deposition modelling. Compos. Part B Eng..

[10] Serra A., González I., Oliver-Ortega H., Tarrès Q., Delgado-Aguilar M., Mutjé P. (2017). Reducing the amount of catalyst in TEMPO-oxidized cellulose nanofibers: Effect on properties and cost. Polymers (Basel).

[11] Isogai A., Saito T., Fukuzumi H. (2011). TEMPO-oxidized cellulose nanofibers. Nanoscale.

[12] Tarrés Q., Boufi S., Mutjé P., Delgado-Aguilar M. (2017). Enzymatically hydrolyzed and TEMPO-oxidized cellulose nanofibers for the production of nanopapers: morphological, optical, thermal and mechanical properties. Cellulose.

[13] Hokkanen S., Bhatnagar A., Sillanpää M. (2016). A review on modification methods to cellulose-based adsorbents to improve adsorption capacity. Water Res..

[14] Habibi Y., Lucia L.A., Rojas O.J. (2010). Cellulose Nanocrystals: Chemistry, Self-Assembly, and Applications. Chem. Rev..

[15] Kim J.H., Shim B.S., Kim H.S., Lee Y.J., Min S.K., Jang D., Kim J. (2015). Review of nanocellulose for sustainable future materials. Int. J. Precis. Eng. Manuf. Green Technol..

[16] Delgado Aguilar M., González Tovar I., Tarrés Farrés Q., Alcalà Vilavella M., Pèlach Serra M.À., Mutjé Pujol P. (2015). Approaching a Low-Cost Production of Cellulose Nanofibers for Papermaking Applications. BioResources.

[17] Henriksson M., Henriksson G., Berglund L.A., Lindström T. (2007). An environmentally friendly method for enzyme-assisted preparation of microfibrillated cellulose (MFC) nanofibers. Eur. Polym. J..

[18] Saito T., Isogai A. (2004). TEMPO-mediated oxidation of native cellulose. The effect of oxidation conditions on chemical and crystal structures of the water-insoluble fractions. Biomacromolecules.

[19] Filipova I., Fridrihsone V., Cabulis U., Berzins A. (2018). Synthesis of Nanofibrillated Cellulose by Combined Ammonium Persulphate Treatment with Ultrasound and Mechanical Processing. Nanomaterials.

[20] Visanko M., Sirviö J.A., Piltonen P., Sliz R., Liimatainen H., Illikainen M. (2017). Mechanical fabrication of high-strength and redispersible wood nanofibers from unbleached groundwood pulp. Cellulose.

[21] Kumagai A., Endo T., Adachi M. (2019). Evaluation of Cellulose Nanofibers by Using Sedimentation Method. JAPAN TAPPI J..

[22] Boufi S., González I., Delgado-Aguilar M., Tarrès Q., Pèlach M.À., Mutjé P. (2016). Nanofibrillated cellulose as an additive in papermaking process: A review. Carbohydr. Polym..

[23] Dimic-Misic K., Gane P.A.C., Paltakari J. (2013). Micro and nanofibrillated cellulose as a rheology modifier additive in CMC-containing pigment-coating formulations. Ind. Eng. Chem. Res..

[24] Lu Y., Chen S.C. (2004). Micro and nano-fabrication of biodegradable polymers for drug delivery. Adv. Drug Deliv. Rev..

[25] Eichhorn S.J., Dufresne A., Aranguren M., Marcovich N.E., Capadona J.R., Rowan S.J., Weder C., Veigel S. (2010). Review: Current international research into cellulose nanofibres and nanocomposites. J. Mater. Sci..

[26] Aitomäki Y., Oksman K. (2014). Reinforcing efficiency of nanocellulose in polymers. React. Funct. Polym..

[27] Fiol N., Vásquez M.G., Pereira M., Tarrés Q., Mutjé P., Delgado-Aguilar M. (2019). TEMPO-oxidized cellulose nanofibers as potential Cu(II) adsorbent for wastewater treatment. Cellulose.

[28] Tarrés Q., Mutjé P., Delgado-Aguilar M. (2019). Towards the development of highly transparent, flexible and water-resistant bio-based nanopapers: tailoring physico-mechanical properties. Cellulose.

[29] Zhang B., Huang C., Zhao H., Wang J., Yin C., Zhang L., Zhao Y. (2019). Effects of cellulose nanocrystals and cellulose nanofibers on the structure and properties of polyhydroxybutyrate nanocomposites. Polymers (Basel).

[30] Abdul Khalil H.P.S., Bhat A.H., Ireana Yusra A.F. (2012). Green composites from sustainable cellulose nanofibrils: A review. Carbohydr. Polym..

[31] Tarrés Q., Saguer E., Pèlach M.A., Alcalà M., Delgado-Aguilar M., Mutjé P. (2016). The feasibility of incorporating cellulose micro/nanofibers in papermaking processes: the relevance of enzymatic hydrolysis. Cellulose.

[32] Fujisawa S., Okita Y., Fukuzumi H., Saito T., Isogai A. (2011). Preparation and characterization of TEMPO-oxidized cellulose nanofibril films with free carboxyl groups. Carbohydr. Polym..

[33] Chaker A., Boufi S. (2015). Cationic nanofibrillar cellulose with high antibacterial properties. Carbohydr. Polym..

[34] Tarrés Q., Oliver-Ortega H., Boufi S., Àngels Pèlach M., Delgado-Aguilar M., Mutjé P. (2020). Evaluation of the fibrillation method on lignocellulosic nanofibers production from eucalyptus sawdust: A comparative study between high-pressure homogenization and grinding. Int. J. Biol. Macromol..

[35] Lavoine N., Bergström L. (2017). Nanocellulose-based foams and aerogels: Processing, properties, and applications. J. Mater. Chem. A.

[36] Osong S.H., Norgren S., Engstrand P. (2016). Processing of wood-based microfibrillated cellulose and nanofibrillated cellulose, and applications relating to papermaking: A review. Cellulose.

[37] Arola S., Malho J.M., Laaksonen P., Lille M., Linder M.B. (2013). The role of hemicellulose in nanofibrillated cellulose networks. Soft Matter.

[38] Pei A., Butchosa N., Berglund L.A., Zhou Q. (2013). Surface quaternized cellulose nanofibrils with high water absorbency and adsorption capacity for anionic dyes. Soft Matter.

[39] Tan B.K., Ching Y.C., Poh S.C., Abdullah L.C., Gan S.N. (2015). A review of natural fiber reinforced poly(vinyl alcohol) based composites: Application and opportunity. Polymers (Basel).

[40] Qiu K., Netravali A.N. (2012). Fabrication and characterization of biodegradable composites based on microfibrillated cellulose and polyvinyl alcohol. Compos. Sci. Technol..

[41] Vilaseca F., Valadez-Gonzalez A., Herrera-Franco P.J., Pèlach M.À., López J.P., Mutjé P. (2010). Biocomposites from abaca strands and polypropylene. Part I: Evaluation of the tensile properties. Bioresour. Technol..

[42] Espinosa E., Bascón-Villegas I., Rosal A., Pérez-Rodríguez F., Chinga-Carrasco G., Rodríguez A. (2019). PVA/(ligno)nanocellulose biocomposite films. Effect of residual lignin content on structural, mechanical, barrier and antioxidant properties. Int. J. Biol. Macromol..

[43] Cinelli P., Chiellini E., Lawton J.W., Imam S.H. (2006). Properties of injection molded composites containing corn fiber and poly(vinyl alcohol). J. Polym. Res..

[44] Peresin M.S., Habibi Y., Vesterinen A.H., Rojas O.J., Pawlak J.J., Seppälä J.V. (2010). Nanofiber Composites of Polyvinyl Alcohol and Cellulose Nanocrystals: Manufacture and Characterization. Biomacromolecules.

[45] Liu D., Sun X., Tian H., Maiti S., Ma Z. (2013). Effects of cellulose nanofibrils on the structure and properties on PVA nanocomposites. Cellulose.

[46] Lee K.Y., Aitomäki Y., Berglund L.A., Oksman K., Bismarck A. (2014). On the use of nanocellulose as reinforcement in polymer matrix composites. Compos. Sci. Technol..

[47] Cherian B.M., Leão A.L., De Souza S.F., Costa L.M.M., De Olyveira G.M., Kottaisamy M., Thomas S. (2011). Cellulose nanocomposites with nanofibres isolated from pineapple leaf fibers for medical applications. Carbohydr. Polym..

[48] Granda L.A., Espinach F.X., Tarrés Q., Méndez J.A., Delgado-Aguilar M., Mutjé P. (2016). Towards a good interphase between bleached kraft softwood fibers and poly(lactic) acid. Compos. Part B Eng..

[49] Espinach F.X., Boufi S., Delgado-Aguilar M., Julián F., Mutjé P., Méndez J.A. (2018). Composites from poly(lactic acid) and bleached chemical fibres: Thermal properties. Compos. Part B Eng..

[50] Goriparthi B.K., Suman K.N.S., Mohan Rao N. (2012). Effect of fiber surface treatments on mechanical and abrasive wear performance of polylactide/jute composites. Compos. Part A Appl. Sci. Manuf..

[51] Thomason J.L. (2002). Micromechanical parameters from macromechanical measurements on glass reinforced polypropylene. Compos. Sci. Technol..

[52] Thomason J.L. (2002). The influence of fibre length and concentration on the properties of glass fibre reinforced polypropylene: 5. Injection moulded long and short fibre PP. Compos. Part A Appl. Sci. Manuf..

[53] Granda L.A., Espinach F.X., López F., García J.C., Delgado-Aguilar M., Mutjé P. (2016). Semichemical fibres of Leucaena collinsii reinforced polypropylene: Macromechanical and micromechanical analysis. Compos. Part B Eng..

[54] Bhatnagar A. (2005). Processing of Cellulose Nanofiber-reinforced Composites. J. Reinf. Plast. Compos..

[55] Tarrés Q., Vilaseca F., Herrera-Franco P.J., Espinach F.X., Delgado-Aguilar M., Mutjé P. (2019). Interface and micromechanical characterization of tensile strength of bio-based composites from polypropylene and henequen strands. Ind. Crops. Prod..

[56] Nakagaito A.N., Iwamoto S., Yano H. (2005). Bacterial cellulose: The ultimate nano-scalar cellulose morphology for the production of high-strength composites. Appl. Phys. A Mater. Sci. Process.

[57] Page D.H. (1969). A theory for the tensile strength of paper. Tappi.

[58] López J.P., Méndez J.A., Mansouri NEEl Mutjé P., Vilaseca F. (2011). Mean intrinsic tensile properties of stone groundwood fibers from softwood. BioResources.

[59] Gurnagul N., Page D. (1989). The difference between dry and rewetted zero-span tensile strength of paper. Tappi J..

[60] Hägglund R., Gradin P.A., Tarakameh D. (2004). Some aspects on the zero-span tensile test. Exp. Mech..

[61] Wathén R., Rosti J., Alava M., Salminen L., Joutsimo O. (2018). Fiber strength and zero-span strength statistics—Some considerations. Nord. Pulp. Pap. Res. J..

[62] Zare Y. (2015). A simple technique for determination of interphase properties in polymer nanocomposites reinforced with spherical nanoparticles. Polymer.

[63] Lizundia E., Delgado-Aguilar M., Mutjé P., Fernández E., Robles-Hernandez B., de la Fuente M.R.M.R., León L.M. (2016). Cu-coated cellulose nanopaper for green and low-cost electronics. Cellulose.

[64] Shinoda R., Saito T., Okita Y., Isogai A. (2012). Relationship between Length and Degree of Polymerization of TEMPO-Oxidized Cellulose Nanofibrils. Biomacromolecules.

[65] Kelly A., Tyson W.R. (1965). Tensile properties of fibre-reinforced metals-copper/tungsten and copper/molybdenum. J. Mech. Phys. Solids.

[66] Vallejos M.E., Espinach F.X., Julián F., Torres L., Vilaseca F., Mutjé P. (2012). Micromechanics of hemp strands in polypropylene composites. Compos. Sci. Technol..

[67] Baxter S.C., Burrows B.J., Fralick B.S. (2016). Mechanical percolation in nanocomposites: Microstructure and micromechanics. Probabilistic Eng. Mech..

[68] Tahouneh V., Mosavi Mashadi M., Naei M.H. (2016). Finite element and micromechanical modeling for investigating effective material properties of polymer–matrix nanocomposites with microfiber, reinforced by CNT arrays. Int. J. Adv. Struct. Eng..

[69] Yu J., Lacy T.E., Toghiani H., Pittman C.U., Hwang Y. (2011). Classical micromechanics modeling of nanocomposites with carbon nanofibers and interphase. J. Compos. Mater..

[70] Li Y., Pickering K.L., Farrell R.L. (2009). Determination of interfacial shear strength of white rot fungi treated hemp fibre reinforced polypropylene. Compos. Sci. Technol..

[71] Tarrés Q., Oliver-Ortega H., Espinach F.X., Mutjé P., Delgado-Aguilar M., Méndez J.A. (2019). Determination of mean intrinsic flexural strength and coupling factor of natural fiber reinforcement in polylactic acid biocomposites. Polymers (Basel).

[72] Pukánszky B. (1990). Influence of interface interaction on the ultimate tensile properties of polymer composites. Composites.

[73] Chopra S., Deshmukh K.A., Peshwe D. (2017). Theoretical prediction of interfacial properties of PBT/CNT nanocomposites and its experimental evaluation. Mech. Mater..

[74] Yeh M.K., Tai N.H., Liu J.H. (2006). Mechanical behavior of phenolic-based composites reinforced with multi-walled carbon nanotubes. Carbon.

[75] Del Rey R., Serrat R., Alba J., Perez I., Mutje P., Espinach F.X. (2017). Effect of sodium hydroxide treatments on the tensile strength and the interphase quality of hemp core fiber-reinforced polypropylene composites. Polymers (Basel).

[76] Arao Y., Fujiura T., Itani S., Tanaka T. (2015). Strength improvement in injection-molded jute-fiber-reinforced polylactide green-composites. Compos. Part B Eng..

[77] Tarrés Q., Soler J., Rojas-Sola J.I., Oliver-Ortega H., Julián F., Espinach F.X., Delgado-Aguilar M. (2019). Flexural properties and mean intrinsic flexural strength of old newspaper reinforced polypropylene composites. Polymers (Basel).

[78] Siqueira G., Bras J., Dufresne A. (2009). Cellulose Whiskers versus Microfibrils: Influence of the Nature of the Nanoparticle and its Surface Functionalization on the Thermal and Mechanical Properties of Nanocomposites. Biomacromolecules.

[79] Liu D., Zhong T., Chang P.R., Li K., Wu Q. (2010). Starch composites reinforced by bamboo cellulosic crystals. Bioresour. Technol..

[80] Siqueira G., Bras J., Follain N., Belbekhouche S., Marais S., Dufresne A. (2013). Thermal and mechanical properties of bio-nanocomposites reinforced by Luffa cylindrica cellulose nanocrystals. Carbohydr. Polym..

[81] Puccini M., Seggiani M., Vitolo S. (2015). Polyethylene and Hydrolyzed Collagen Blend Films Produced by Blown Extrusion. Chem. Eng. Trans..

[82] Zafar M.T., Maiti S.N., Ghosh A.K. (2016). Effect of surface treatments of jute fibers on the microstructural and mechanical responses of poly(lactic acid)/jute fiber biocomposites. RSC Adv..

[83] Campano C., Merayo N., Balea A., Tarrés Q., Delgado-Aguilar M., Mutjé P., Blanco Á. (2018). Mechanical and chemical dispersion of nanocelluloses to improve their reinforcing effect on recycled paper. Cellulose.

[84] Nakagaito A.N., Yano H. (2005). Novel high-strength biocomposites based on microfibrillated cellulose having nano-order-unit web-like network structure. Appl. Phys. A Mater. Sci. Process..

[85] Alcalá M., González I., Boufi S., Vilaseca F., Mutjé P. (2013). All-cellulose composites from unbleached hardwood kraft pulp reinforced with nanofibrillated cellulose. Cellulose.

[86] Célino A., Fréour S., Jacquemin F., Casari P. (2014). The hygroscopic behavior of plant fibers: A review. Front. Chem..

[87] Sehaqui H., Zhou Q., Berglund L.A. (2011). Nanostructured biocomposites of high toughness—A wood cellulose nanofiber network in ductile hydroxyethylcellulose matrix. Soft Matter.

[88] Colom X., Carrasco F., Pages P., Canavate J. (2003). Effects of different treatments on the interface of HDPE / lignocellulosic fiber composites. Compos. Sci. Technol..

[89] Oliver-Ortega H., Granda L.A., Espinach F.X., Mendez J.A., Julian F., Mutjé P. (2016). Tensile properties and micromechanical analysis of stone groundwood from softwood reinforced bio-based polyamide11 composites. Compos. Sci. Technol..

[90] Jiménez A.M., Espinach F.X., Delgado-Aguilar M., Reixach R., Quintana G., Fullana-i-Palmer P., Mutjé P. (2016). Starch-based biopolymer reinforced with high yield fibers from sugarcane bagasse as a technical and environmentally friendly alternative to high density polyethylene. BioResources.

[91] Shah D.U., Nag R.K., Clifford M.J. (2016). Why do we observe significant differences between measured and ‘back-calculated’ properties of natural fibres?. Cellulose.

[92] Zare Y. (2015). Effects of interphase on tensile strength of polymer/CNT nanocomposites by Kelly-Tyson theory. Mech. Mater..

[93] Salem S., Oliver-Ortega H., Espinach F.X., Hamed KBen Nasri N., Alcalà M., Mutjé P. (2019). Study on the Tensile Strength and Micromechanical Analysis of Alfa Fibers Reinforced High Density Polyethylene Composites. Fibers Polym.

[94] Delgado-Aguilar M., Julián F., Tarrés Q., Méndez J.A., Mutjé P., Espinach F.X. (2017). Bio composite from bleached pine fibers reinforced polylactic acid as a replacement of glass fiber reinforced polypropylene, macro and micro-mechanics of the Young’s modulus. Compos. Part B Eng..

